# Periprosthetic Transpatellar Fracture Replacement With Autogenous Iliac Graft: A Technical Note

**DOI:** 10.7759/cureus.64394

**Published:** 2024-07-12

**Authors:** Konstantinos Zygogiannis, Dimitrios Koulalis, Dimitrios Kalatzis, Georgios C Thivaios

**Affiliations:** 1 Scoliosis and Spine Department, KAT Hospital, Athens, GRC; 2 Orthopedics and Traumatology, Attikon University Hospital, Athens, GRC; 3 Orthopedics and Traumatology, Laiko General Hospital of Athens, Athens, GRC

**Keywords:** total knee arthroplasty, surgical technique, iliac autograft, patella reconstruction, tka revision, complex patella fracture

## Abstract

Periprosthetic fractures involving total knee arthroplasty (TKA) components, particularly involving the patella, can present a significant challenge regarding orthopedic surgery. This technical note outlines an approach for the reconstruction of complicated periprosthetic transpatellar fractures, with poor bone stock, utilizing autogenous iliac graft. This kind of procedure requires careful preoperative evaluation of imaging, precise intraoperative planning, and strict postoperative management to achieve adequate postoperative results. The inventive option of utilizing an autogenous iliac graft for reconstruction suggests its potential benefits in addressing the unique biomechanical demands of patellar fractures in TKA patients with poor bone stock. Key technical aspects of this approach are highlighted and include graft harvest, graft preparation, and fixation techniques. Overall, this technique can provide a golden standard bailout for periprosthetic transpatellar fracture reconstruction and potentially offer orthopedic surgeons a comprehensive framework for addressing this challenging clinical scenario.

## Introduction

Periprosthetic fractures, especially those involving the patella, represent a rare but rigorous complication following total knee arthroplasty (TKA). First reported in the early 1970s, these fractures have posed a significant challenge to orthopedic surgeons due to the complexity of their management, the high failure rates, and their potential to compromise the functional outcomes of TKA procedures [1]. Throughout the evolution of TKA techniques, the incidence of periprosthetic fractures has remained relatively low, on average 1.19%, but continues to be a topic of concern, especially with the increasing prevalence of TKA procedures and their revisions worldwide [2]. Excision of the patella is sometimes indicated for intractable pain due to patellofemoral arthrosis or following severe comminution from fractures [3]. However, extension lag and quadriceps weakness are known complications of a patellectomy [4]. Additionally, subluxation of the extensor apparatus may occur. If a total joint replacement becomes necessary post-patellectomy, instability can arise if a non-constrained system is utilized [5]. Patellar reconstruction enables the restoration of the moment arm, thereby improving quadriceps leverage and allowing for patellar resurfacing. Various methods have been described for total and partial patella reconstruction using autogenous and allogenic bone [6,7]. In this technical note, we aim to provide useful step-by-step information concerning the reconstruction with autogenous iliac autograft, emphasizing key features during the surgical approach.

## Technical report

Surgical technique

Preoperative Planning

**Figure 1:**
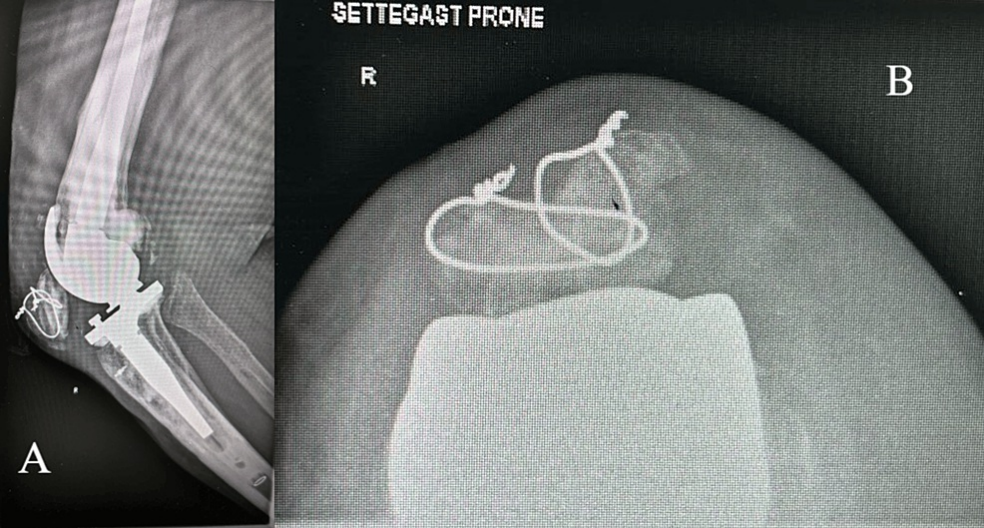
Preoperative X-rays of the knee demonstrating the periprosthetic patella fracture. (A) Lateral view of the knee. (B) Settegast prone view of the patella.

Prior to surgery, detailed preoperative planning is essential to ensure optimal outcomes. This includes careful assessment of the patient's medical history (infection, osteoporosis, diabetes, vascular pathology, and other comorbidities), imaging studies (such as standing, flexion/extension X-rays, and computed tomography scans), and evaluation of the extent of the periprosthetic fracture (Figure 1). Surgical approach selection is based on fracture location, implant type, and patient-specific factors. A written patient consent form was obtained from the patient to publish intraoperative pictures and the case as a scientific paper.

Graft Harvest

**Figure 2:**
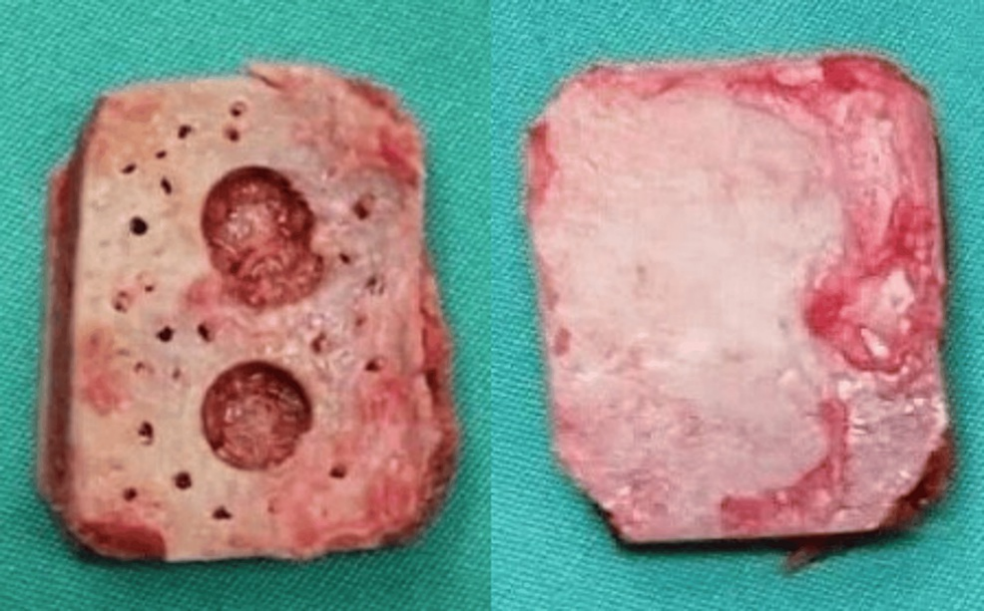
Iliac crest autograft after preparation technique.

**Figure 3:**
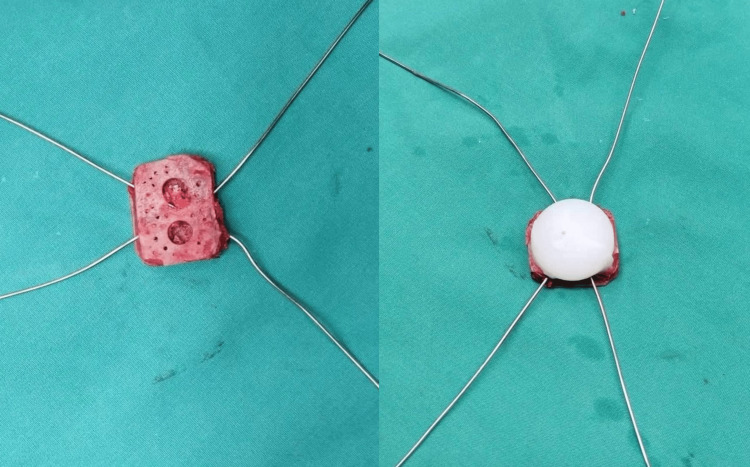
X-cross insertion of wires along with the cemented polyethylene.

The surgical procedure begins with the harvesting of the autogenous iliac graft. The patient is placed in a supine position, and a standard incision is made over the anterior iliac crest. Care is taken to preserve the integrity of the surrounding soft tissues to avoid intraoperative injury to neurovascular structures and a postoperative hematoma. A bone graft is then harvested from the iliac crest using an osteotome or oscillating saw. Simple bone wax can be used to stop the blood oozing from the bony structures. The size and shape of the graft are tailored to match the patella in cases of complete loss of the bony surface, ensuring optimal graft-host interface and stability. The preparation technique includes multiple drilling of the surface that will come in contact with cement and polyethylene to increase the bone-cement interface and another two holes according to the polyethylene model, as shown in Figure 2. The next step includes the X-cross insertion of wires that will serve as internal fixation of the autograft (Figure 3).

Fracture Reduction and Preparation

Next, the surgical site is prepared for fracture reduction and graft placement. The periprosthetic fracture is carefully reduced using K-wires or clumps under direct visualization, restoring anatomical alignment and ensuring a proper position of the fracture fragments. Any fibrous tissue or hematoma around the fracture site is carefully debrided to promote optimal bone healing and decrease the risk of pseudarthrosis.

Graft Insertion and Fixation

**Figure 4:**
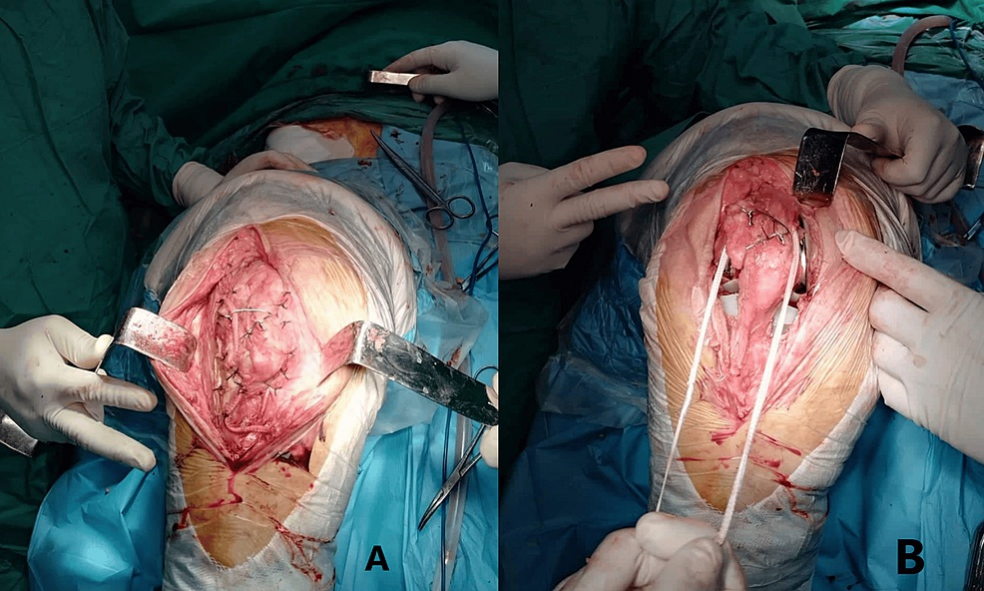
Intraoperative image of patellar replacement. (A) Intraoperative look after patella and soft tissue reconstruction. (B) Use of fiber tape for additional soft tissue support.

The autogenous iliac graft is then inserted into the fracture site to provide structural support. The graft is positioned to fill the defect created by the fracture and to achieve stable fixation. Depending on the fracture pattern and implant stability, additional fixation may be required, such as the use of screws or cerclage wires to secure the graft in place or ligamentous reinforcement (Figure 4A). In this case, we made use of fiber tape from Arthrex (Naples, FL) for additional soft tissue support and stability (Figure 4B). Care is taken to avoid interference with the existing TKA components and to preserve patellar tracking and joint biomechanics.

Closure and Postoperative Management

Once graft placement and fixation are complete, the surgical site is thoroughly irrigated, and hemostasis is achieved. The wound is closed in layers, and a sterile dressing is applied. Postoperative management includes early controlled mobilization and physical therapy to promote joint range of motion and graft incorporation. Close monitoring for signs of graft loosening, such as radiographic evidence is essential to assess the progress and ensure long-term implant stability.

## Discussion

Periprosthetic fractures around the components of TKA, particularly involving the patella, are not that frequent and can come with significant implications regarding postoperative outcomes. While the overall occurrence of such fractures varies depending on the studied population and duration of follow-up, patellar fractures play a notable role in these occurrences. Various studies reported that the incidence rate of patellar fractures post-TKA can range between 0.3% and 2%, while an elevated risk incidence is observed among specific patient cohorts, such as those with rheumatoid arthritis or a history of patellar resurfacing [8]. Smith et al., in a retrospective analysis of patients, observed a substantial rate of fracture healing and favorable clinical outcomes, characterized by a notable improvement in knee function and pain alleviation post surgery [9]. Hozack et al. demonstrated favorable outcomes using nonoperative approaches for patients with nondisplaced fractures that required an intact extensor mechanism. However, they observed unsatisfactory results, even with surgical intervention, in cases where there was disruption to the extensor mechanism. This highlights the importance of considering the integrity of the extensor mechanism when determining the appropriate treatment [10].

Successful functional outcomes are frequently observed in patients who lack extension lag and possess sufficient bone stock [11]. On the contrary, unfavorable outcomes might be linked to osteoporosis, particularly in elderly female patients with rheumatoid arthritis. Adhering to the fundamental principles of TKA is crucial to prevent periprosthetic patellar fractures (PPPFs), which requires proper alignment of the extensor mechanism, soft tissue balance, and precise bone cuts [12]. Type II PPPFs are associated with a high incidence of complications (50%) and a need for revision surgeries (42%) following osteosynthesis. Rehabilitation plays a crucial role in achieving preoperative levels of activity. Leaving an osseous shell when removing the patella component may contribute to anterior knee discomfort and crepitus [13]. Repairing only the extensor mechanism can often lead to poor outcomes.

In a variety of studies on patellar reconstruction techniques using autogenous or allogenic bone, according to radiographic data, a reduction in patellar thickness is observed postoperatively, ranging from 2.3 to 9.0 mm [14]. Those findings are consistent with showing an average thickness reduction of 4.2 mm during the follow-up. As a result, it is recommended that the reconstructed patella should have a minimum thickness of 10 mm during the procedure [15]. Despite the quality of reduction, it is considered that partial bone resorption does not negatively impact clinical outcomes. Additionally, it is believed that creating a "new" patella with an autogenic or allogenic bone graft is an effective surgical method to improve knee function and alleviate typical complaints following a patellectomy. Direct comparison between available methods with accurate conclusions is challenging due to the limited size of patient series or case reports available. Each technique has its unique advantages and potential issues, such as high failure rates, postoperative infection, or low satisfaction rates.

## Conclusions

In conclusion, periprosthetic transpatellar fractures pose unique challenges in the field of orthopedic surgery, requiring careful consideration of patient-specific factors and meticulous surgical technique. The utilization of an autogenous iliac graft represents a promising approach for reconstructing these fractures.
